# Temporal Effects of Cigarette Smoke and Phytochemical-Based E-Liquid Aerosols on Tracheo-Alveolar Histopathology and the IL-6/TNF-α Molecular Signaling Axis

**DOI:** 10.3390/cimb48060618

**Published:** 2026-06-15

**Authors:** Awal Prasetyo, Dora Maftikhati, Levina Athaya Anarizta, Nazhira Ghina Setyawan, Anindha Waradita Putri Yuwono, Maria Meutia Saleha, Farahdita Ramadhanti Annisa Mukti, Hermawan Istiadi, Udadi Sadhana, Fathur Nur Kholis

**Affiliations:** 1 Department of Biomedical Science, Faculty of Medicine, Universitas Diponegoro, Semarang 50275, Indonesia; doramaftikhati30@gmail.com (D.M.); levinaathaya@gmail.com (L.A.A.); 2 Department of Anatomic Pathology, Faculty of Medicine, Universitas Diponegoro, Semarang 50275, Indonesia; hermawanistiadi@fk.undip.ac.id (H.I.); udadisadhana@gmail.com (U.S.); 3 Faculty of Medicine, Universitas Diponegoro, Semarang 50275, Indonesia; nazhiraghina@gmail.com (N.G.S.); anindhawpy@gmail.com (A.W.P.Y.); mariameutia@gmail.com (M.M.S.); farahdita2002@gmail.com (F.R.A.M.); 4 Division of Pulmonology, Department of Internal Medicine, Kariadi Hospital, Faculty of Medicine, Universitas Diponegoro, Semarang 50275, Indonesia; drfnkholis@gmail.com

**Keywords:** cigarette smoke, e-nicotine aerosol, ascorbic acid aerosol, IL-6, TNF-α, SOD-3, MDA, tracheo-alveolar histopathology

## Abstract

This study compared the temporal effects of traditional cigarettes and e-cigarettes on lung health in male *Rattus norvegicus* over 8- and 12-week periods. Thirty rats were evaluated for tracheal/alveolar histopathology and systemic markers (IL-6, TNF-α, SOD-3, MDA). Chronic cigarette exposure (12 weeks) and nicotine aerosol (8 weeks) significantly suppressed weight gain, while ascorbic acid aerosol caused less growth inhibition. At 8 weeks, cigarette exposure (K3) induced adaptive tracheal mucosal thickening (66.88 ± 17.92 µm vs. 52.40 ± 2.63 µm in control K1), increased goblet cells (4.2 ± 2.44 N/mm), elevated SOD-3 (12.75 ± 1.10 pg/mL), and initiated emphysematous alveolar expansion (469.77 ± 91.31 µm vs. 202.03 ± 29.38 µm in K1 in K1). Conversely, 12-week cigarette smoke (K4) triggered epithelial exhaustion, significantly thinning the tracheal mucosa (34.65 ± 6.55 µm) and elevating systemic IL-6 (11.45 ± 1.17 pg/mL vs. 8.43 ± 0.88 pg/mL in control K2). Notably, chronic electronic ascorbic acid aerosolization (K6) preserved localized alveolar structural layouts and limited septal thickening compared with nicotine groups. However, it failed to suppress systemic inflammation, as evidenced by elevated IL-6 levels. In conclusion, while ascorbic acid aerosols moderate localized parenchymal destruction compared to nicotine, chronic aerosol exposure accelerates systemic immune activation.

## 1. Introduction

The rising number of smokers is a global challenge. Smoking devices have become more varied, including traditional cigarettes and electronic cigarettes (e-cigs) or aerosols, commonly referred to as vapes. In 2020, about 1.18 billion people smoked tobacco [1], while an estimated 68 million used vapes [2,3], with an additional 82 million users worldwide in 2021 [4]. Both smoking and vaping cause serious issues, including lung damage, heart disease, and addiction. Vapes release harmful substances, including nicotine, tiny particles, heavy metals, and chemicals, and are a dangerous alternative to cigarettes [5,6,7]. Other harmful substances are present in e-liquids heated with humectants, flavorings, propylene glycol, glycerin, and aldehyde, which become aerosolized and later cause notable harm [8,9]. Smoking, whether through cigarettes or vapes, can lead to long-term health issues, including increased oxidative stress, airway inflammation, immune suppression, and impaired alveolarization [10,11].

E-cigarette aerosol harms the trachea and lungs by causing inflammation and damaging the airway. Small particles penetrate deep into the lungs, while larger ones settle in the trachea. This injury leads to significant lung changes due to chemical damage and inflammation. Key effects include the accumulation of lipid-laden macrophages, the death of lung cells, and increased neutrophilic inflammation. As a result, the alveolar walls become thicker, the alveolar structure is damaged, and more inflammatory cells invade the area [12,13]. Ingredients like propylene glycol and vegetable glycerin dry out the respiratory lining, making it harder to clear mucus. High-wattage heating of e-liquids creates harmful chemicals that damage cell membranes and weaken airway function. Both e-cigarette aerosol and cigarette smoke can harm the trachea and alveoli through inflammation and cell toxicity [14,15].

Aerosols weaken connections between epithelial cells in the airways, damaging the airway surface. Toxic substances kill these cells, leading to shedding and exposing the underlying layer. This injury causes inflammation, as damaged cells release signals such as IL-1β, IL-6, and IL-8, which attract neutrophils and macrophages. These produce Tumor Necrosis Factor-alpha (TNF-α) and reactive oxygen species (ROS), which further exacerbate inflammation. Additionally, levels of pro-inflammatory cytokines such as IL-6 and TNF-α increase in the lungs. Studies show that even short-term exposure to flavored e-cigarette aerosol can cause lung inflammation, while longer exposure can maintain this inflammation, harm lung structure, and reduce lung function. E-cigarette chemicals also irritate the trachea and worsen inflammation [16,17,18].

The “harm reduction paradox” of e-cigarettes highlights a conflict: they can help smokers quit burning tobacco and reduce harm, but they may also create a new generation of nicotine-addicted young users who might not have smoked otherwise, which increases harm [19]. In addition, many people think that using ascorbic acid (vitamin C) or fruit-flavored liquids is a healthier alternative to smoking. These products claim to provide a “wellness” experience and antioxidant benefits, serving as a safe way to satisfy the hand-to-mouth habit without nicotine addiction [20]. The hypothesis that inhaling ascorbic acid (AA) may support the immune system in the nose and sinuses and reduce inflammation, especially in individuals with asthma or breathing problems [21], has yet to be proven. The antioxidant property of AA could reduce ROS levels in the body and prevent lipid peroxidation through electron transfer [22,23], but it may not have the same effect when delivered as an aerosol, and the research on the safety and toxicity of inhaling AA is limited. This presumption is largely unproven, with significant research indicating potential risks rather than health benefits and creating a complex public health challenge [24].

Common markers for assessing oxidative stress included superoxide dismutase-3 (SOD-3) and malondialdehyde (MDA). SOD-3 is an extracellular enzyme that can suppress the inflammatory response. SOD-3 has been shown to maintain ECM structure, reduce oxidative stress in macrophages, and increase lung space [25,26]. MDA is a lipid peroxide marker used to assess oxidative stress and redox signaling. Elevated MDA levels indicate increased lipid peroxidation. Lipid peroxidation is highly linked to the amount of ROS, whereas lipid peroxidation is susceptible to oxidation by free radicals. MDA is considered a reliable marker of oxidative stress due to its reactivity and toxicity [27,28,29,30].

Chemical aerosol agents cause microscopic damage to the trachea, leading to reduced ciliary activity and increased goblet cell numbers, which results in mucosal hypersecretion. This disrupts the NMC defense mechanism that protects the respiratory tract. Both vapes and cigarettes harm tracheal histology, with mucus trapping harmful substances and stimulating goblet cell proliferation. This increase in goblet cells elevates mucin expression and thickens the mucosal layer, ultimately reducing the effectiveness of the NMC defense [31,32,33]. Smoking, whether through cigarettes or vapes, harms the body in different ways [34,35,36], and the duration of exposure. Nicotine is a common e-liquid ingredient, and researchers are exploring the use of vitamins as alternatives [37].

This study will compare different exposure times and the effects of nicotine and AA in vaping versus cigarette smoke. It will measure plasma levels of pro-inflammatory cytokines (IL-6, TNF-α), oxidative stress markers (SOD-3, MDA), goblet cell counts, tracheal epithelial thickness, and alveolar–pulmonary changes. This study investigates two key areas: first, it provides evidence on whether inhaling nicotine-free and phytochemical aerosols, such as ascorbic acid, constitute a valid harm-reduction strategy or pose respiratory risks. Second, it analyzes airway remodeling at 8 versus 12 weeks and systemic markers (IL-6, TNF-α, SOD-3, MDA) to better understand the transition from early tracheal changes to chronic alveolar damage, aiming to inform diagnostic and therapeutic protocols for lung injuries associated with alternative smoking devices.

## 2. Materials and Methods

To ensure transparent and accurate reporting of research, this study used the ARRIVE guidelines 2.0 (Animal Research: Reporting In Vivo Experiments) [38].

### 2.1. Study Design

A true experimental randomized post-test-only control group was designed to test causality. Subjects were randomly assigned to experimental or control groups, with the experimental group exposed to cigarette smoke and aerosols (independent variable). Outcomes, including pro-inflammatory cytokines, oxidative stress, and trachea–alveolar histopathology (dependent variables), were measured only after the intervention, ensuring high internal validity. Our selection of the 8-week and 12-week endpoints was a deliberate strategy to capture the definitive “tipping points” of the acute-to-chronic transition phase.

### 2.2. Sample Size

A total of 30 rats were used as subjects; each experimental unit was assigned 5 rats, providing biological replication (*n* = 5) per group. The minimal sample size was calculated using the ‘resource equation’ method (*E* = *Total number of animals* − *Total number of groups*). For our design (6 groups, 5 animals per group), *E* = 30 − 6 = 24, which falls squarely within the robust and scientifically acceptable limits for molecular signaling research and accommodates potential biological variability.

With *E* = 24, the total sample size is robust and generally considered highly acceptable in molecular signaling research (IL-6/TNF-α), where biological variability can be high or “drop-outs” during the smoke-exposure phase might occur. The likelihood of detecting an effect, if one exists, for “all-or-nothing” or “large” morphological changes in tracheo-alveolar tissue with *n* = 5 per group was quantified as “Power” (1 − *β*), achieving an approximate power of 80%, predicated on a large effect size (d ≥ 1.5).

### 2.3. Inclusion and Exclusion Criteria

Animals were included in the study if they successfully completed the intervention. Rats were excluded for excessive weight loss (>20%), underlying illness, motor impairments affecting behavior, unexpected injuries, or death during the study.

### 2.4. Grouping and Randomization

All rats were divided into control groups without exposure, with two durations: 8 and 12 weeks (K1 and K2). The four other treatment groups had different durations and aerosol exposures: three cigarettes smoked/day for 8 weeks (K3) and 12 weeks (K4); e-nicotine liquid-based vape aerosol for 8 weeks (K5); and e-ascorbic acid liquid-based vape aerosol for 12 weeks (K6).

Simple random sampling allocated subjects to control and treatment groups using a computerized random-number generator to select from a numbered list of rats, giving each an equal chance of selection. To reduce environmental confounders, cages were randomly assigned positions on the rack, controlling for local variations in light and temperature. Additionally, tissue collection and plasma harvesting were conducted in a randomized order to avoid the effects of time of day or processing sequence.

Before and after a week of acclimatization, all subjects were weighed to ensure that each treatment group was balanced for body weight, controlling for potential confounding differences. All procedures (weighing, aerosol exposure, and blood sampling) were conducted by the same person, or the investigators were blinded to treatment groups during testing to prevent observation bias.

### 2.5. Blinding

The animal caretakers and data analysts were blinded to the stages (i.e., allocation, intervention, or outcome assessment). They were unaware of group assignments, which were coded to prevent conscious or subconscious bias in data collection and analysis.

### 2.6. Statistical Methods

Statistical analysis was performed using IBM SPSS Statistics 25, employing a one-way analysis of variance (ANOVA) followed by Tukey’s HSD to assess differences between experimental groups. This specific combination of tests was used to compare the averages of three or more groups to see the differences in histopathological changes in the trachea–mucosa and alveolus, as well as IL-6 and TNF-α, and SOD-3 and MDA levels, as dependent variables measured over time in the same subjects, controlling for type I error inflation while identifying specific differences.

### 2.7. Experimental Animals

A total of 30 specific-pathogen-free (SPF) male Wistar strain (*Rattus norvegicus*) rats, 8 weeks old and weighing 160–180 g, were utilized. The Wistar rats used have a standardized, outbred genetic background, bred specifically for scientific purposes in the Biology Laboratory at Semarang State University. Housing and treatment under experimental conditions were managed at the Animal Laboratory of the Faculty of Medicine, Universitas Diponegoro, Semarang, Indonesia.

### 2.8. Acclimatization

All rats in each experimental group of five were maintained in a stable room with a 12 h light–dark cycle, temperatures between 22 and 26 °C, an appropriate humidity of 40–60%, and a stimulating, clean, spacious environment with dim, indirect light and good air circulation, but no direct draught. For one week, the rats underwent an acclimatization period and were given standard feed A594K pellet and water ad libitum. All subjects were weighed using a pharmacologic digital scale (0.1–5000 g) before treatment.

### 2.9. Experimental Procedures

A cigarette smoke chamber and an electronic cigarette aerosol chamber were custom-made and prepared following the protocol of the University of Michigan Institutional Animal Care and Use Committee (IACUC) [39].

#### 2.9.1. Cigarette Smoke Exposure

Local filtered clove cigarettes (kretek) from PTJ Batch 2024 (Kudus, Indonesia) were used in 18 L, semi-closed exposure chambers to simulate second-hand smoke exposure. Each cigarette contains 32 mg of tar, 1.8 mg of nicotine, and 15 mg of carbon monoxide (CO), in accordance with Indonesian standards. Kretek has a shredded tobacco-to-clove ratio of about 60:40 to 70:30, producing 4–5 mg of eugenol during burning. This choice aligns with the consumption trends in Southeast Asia, where clove tobacco is dominant.

The smoke was pushed into the chamber using “syringe method” (a manually replicated “puff” action of a human smoker) with a basic manifold to connect the cigarette to the chamber: the “holder” (a rubber stopper or plastic tube that fits the cigarette filter tightly), the “puff” tool (a large 50 mL plastic syringe), three-way stopcock (allows to draw smoke from the cigarette and then switch the path to push it into the box without disconnecting everything), and inlet port (a small hole in your container box (sealed with a grommet) where the smoke enters).

The exposure time was based on a typical inhalation protocol that involves exposing the rats to the smoke of 1.25 cigarettes over 30 min, twice a day, with a 5 min ventilation period in between to prevent hypoxia. The dosage of cigarettes that was used in this research is equivalent to the dosage of heavy smoking in humans, which is generally defined as smoking 20 or more cigarettes per day (approximately one pack or more). To calculate the equivalent dose for a rat based on human cigarette consumption using the Laurence and Bacharach method, a body-weight-to-surface-area ratio is typically used, with the standard conversion factor from a human (70 kg BW) to a rat (200 g) of 0.018. For adjusting for the larger volume of an 18 L container, here is the calculation for 6 rats (180 g each) to match the exposure of a human smoking 20 cigarettes per day: 0.324 cigarettes per rat, and the total biological requirement for 6 rats was 1.944 cigarettes per day, or 2.33 cigarettes per container per day, or the same as 3 cigarettes for equalization.

#### 2.9.2. Electronic-Vape Aerosol Exposure

An electronic-vape aerosol exposure technique was performed manually to investigate the impact of e-cigarette aerosol (nicotine liquid base and ascorbic acid liquid base) or to measure second-hand exposure in environmental simulations on the tracheal–alveolar system. These techniques involve a controlled, non-automated vape mod to generate aerosol from nicotine or ascorbic acid-containing e-liquid, which is then delivered to an exposure chamber. Aerosolization was performed using a standardized, regulated vape mod equipped with a rebuildable dripping atomizer (RDA) (Shenzhen Jikang Weipu Technology Co., Ltd., Shenzhen, China) with a 0.4 Ω Kanthal A1 coil wrapped with organic cotton as the atomization core. The device was operated at a fixed heating power of 40 W (yielding an output voltage of approximately 4.0 V).

The dosage of nicotine e-liquid-based vape aerosol, corresponding to a high-intensity toxicological dose [40], was set at 0.5 mL daily, with puffs lasting 4 s at 30 s intervals, for a 200 g rat. This regimen significantly stresses the system, approaching the upper limit of human use, thereby facilitating the observation of potential respiratory or vascular damage within a brief 8-week study. Nicotine-based E-liquid: Structured on a standardized 50:50 volume-to-volume ratio of propylene glycol (PG) to vegetable glycerin (VG) (USP-grade ≥ 99.5% purity). It contained 3 mg/mL of pure nicotine freebase without any added cooling agents, colorants, or secondary sweetening additives.

The ascorbic acid dose was set at 0.5 mL/day (100 mg), which was also administered to a treatment group and was approximately equivalent to 4.8 g/day in humans. It was formulated in an identical 50:50 PG/VG carrier vehicle. It was also compounded by dissolving 10 mg/mL of analytical-grade L-ascorbic acid, maintaining an unflavored profile to isolate the pure effect of the aerosolized phytochemical carrier from flavoring chemicals. This is significantly equal to the standard human dietary maximum (2 g) and would be considered a pharmacological or high-dose experimental level.

#### 2.9.3. Euthanasia

At 8 and 12 weeks, rats were euthanized using humane endpoints, and all procedures were conducted in accordance with the American Veterinary Medical Association (AVMA) guidelines. In this research setting, decapitation was performed by trained personnel using well-maintained equipment to preserve cellular stability for accurate measurement of oxidant and antioxidant enzymes.

#### 2.9.4. Preparation of Trachea–Alveolar Specimen for Histological Examination

Harvesting the trachea of a rat involves careful dissection of the cervical region to isolate the airway from surrounding connective tissue and muscles [40]. The tissues were typically fixed in 10% neutral buffered formalin immediately upon removal to prevent autolysis. The fixed tissue was dehydrated through increasing concentrations of alcohol (e.g., 70% to 100%), cleared in xylene, and embedded in paraffin wax. Using a microtome, the paraffin block was cut into thin sections (5 μm) by cross-section to display the horseshoe-shaped cartilage, the lumen, and the posterior trachealis muscle. The staining procedure using the Hematoxylin and Eosin (H&E) technique showed the general morphology (basophilic nuclei and eosinophilic connective tissue). The number of goblet cells and the thickness of the mucosal epithelium were observed using a light microscope at 400× magnification.

Lung tissue was removed through a thoracotomy procedure, then handled, processed, and stained with H&E as standard for histopathological examination. Using a light microscope at 400× magnification (Olympus Corporation, Tokyo, Japan), the alveolar perimeter length, the degree of alveolar wall damage, and the number of inflammatory cells were measured in a blinded manner by two pathologists.

#### 2.9.5. Histopathology Measurements

To ensure technical replication for histopathological measurements, five different randomized fields of view were analyzed for each tracheal ring, along with five separate viewpoints for each lung tissue profile, all of which were cross-evaluated independently by two blinded pathologists. The tracheal preparation was made from a transverse section of the trachea and analyzed with a light microscope at 400× magnification. The number of goblet cells will be measured using the GCD (Goblet Cells Density) method. At the same time, the thickness of the mucosal epithelium will be measured from 5 selected fields of view per tracheal ring. If the entire circumference is visible, researchers often count four specific quadrants (12, 3, 6, and 9 o’clock positions). We used quantitative methods, including Linear Density (cells per mm), which measures the number of goblet cells per unit length of airway. Using imaging software (ImageJ^®^ v.1.54t), the length of the epithelial basement membrane was measured in millimeters (L). The number of cells was the count of all positive goblet cells that touch the basement membrane (N); GCD = N (cell number)/L (length of the basement membrane in millimeters).

The thickness of the tracheal mucosal epithelium was measured linearly using a calibrated ocular micrometer or digital imaging software. Measurements were taken from the apical surface (excluding the cilia) down to the basement membrane. To ensure accuracy, 5 fields of view per tracheal ring transection were measured. Measurements are typically taken at multiple standardized points around the tracheal ring to calculate a mean value, which is recorded in micrometers (µm).

Under a microscope connected to Cellsens Olympus Imaging Software (version 1.17), the circumference of the alveolus was measured at 5 different viewpoints at 400× magnification by two pathologists with µm accuracy. The distribution of inflammatory cells was counted in 100× magnification and in 5 different viewpoints, and subjectively classified into three categories: (1) light distribution of inflammatory cells scored 1; (2) medium distribution of inflammatory cells scored 2; and (3) heavy distribution of inflammatory cells scored 3. The damage of alveolus lining cells viewed in 100× magnification on 5 different views were then categorized based how many damaged alveoli were found on any given viewpoint as follows: (1) no damage scored 0; (2) less than quarter of a total alveolus in each viewpoint scored 1; (3) more than a quarter yet less than a half of a total alveolus in each viewpoint scored 2; (4) more than half up to 75% of a total alveolus in each viewpoint scored 3; and (5) only less than 25% healthy alveoli found scored 4.

#### 2.9.6. IL-6 and TNF-α Examination

For biochemical assays (ELISA for IL-6, TNF-α, SOD-3, and MDA) (Wuhan Elabscience Biotechnology Co., Ltd., Wuhan, China), all plasma samples and standards were run in technical duplicates to calculate mean optical densities and minimize intra-assay coefficient of variation. Blood samples of 5 mL per rat were collected via the retro-orbital sinus into tubes containing EDTA. Samples should be centrifuged at approximately 1000–2000× *g* for 15 min at 4 °C. Plasma must be separated immediately and assayed on the same day to avoid repeated freeze–thaw cycles, which significantly reduce detectable levels of IL-6 and TNF-α. Use Kit ELISA IL-6 (RAT) Elabscience E-EL-R0015 (96 well) and Kit ELISA TNF-α (RAT) Reedbio RE1060R (96 well) (Wuhan Elabscience Biotechnology Co., Ltd., Wuhan, China; Reed Biotech Ltd., Wuhan, China). The mechanical steps of Sandwich ELISA procedures (Add sample → Incubate → Wash → Add Detection → Wash → Add HRP → Wash → Color) are identical for running those kits. Ensure you use the specific Biotinylated Antibody and Standard provided in each respective box—they are not interchangeable between kits.

Before starting, ensure that all reagents and samples reach room temperature (18–25 °C) and that the plasma is not hemolyzed, as released intracellular components can interfere with IL-6 and TNF-α measurements. Dilute the 25× wash buffer concentrate with deionized water to create a 1× working solution. Reconstitute the Rat IL-6/TNF-α standard with the provided Reference Standard & Sample Diluent. Perform a 2-fold serial dilution to create a standard curve (e.g., 1000, 500, 250, 125, 62.5, 31.25, 15.63, and 0 pg/mL). Dilute the 100× concentrate to a 1× working solution using the Biotinylated Detection Ab Diluent (prepare only what is needed for the day). Dilute the 100× HRP Conjugate to 1× using the HRP Conjugate Diluent.

Add 100 μL of the prepared standards, blank, and plasma samples into the appropriate wells. Cover the plate with the sealer and incubate for 90 min at 37 °C (during this phase, the IL-6 in the plasma binds to the capture antibody coated on the plate). Discard the liquid from each well. Immediately add 100 μL of the 1× Biotinylated Detection Ab working solution to each well. Incubate for 1 h at 37 °C. This antibody binds to a different epitope on the IL-6/TNF-α molecule, forming the “sandwich”. Aspirate the liquid from each well and add 350 μL of wash buffer (first wash). Let it soak for 1–2 min, then discard the liquid and pat the plate dry against clean absorbent paper. Repeat this process 3 times. Add 100 μL of 1× HRP Conjugate working solution to each well. Incubate for 30 min at 37 °C (the Streptavidin-HRP binds to the biotin on the detection antibody). Aspirate and wash the wells 5 times (second wash) using the same process described in Step 3. This step is critical for removing any unbound HRP, which could lead to high background noise. Add 90 μL of substrate reagent (TMB) to each well. Cover the plate and incubate for about 15 min at 37 °C. Protect the plate from light. The solution will turn blue in wells containing IL-6/TNF-α. Add 50 μL of Stop Solution to each well. The color will immediately change from blue to yellow. Immediately measure the optical density (OD) of each well at 450 nm using a microplate reader (BioTek ELx800 Absorbance Microplate Reader, Agilent Technologies, Santa Clara, CA, USA).

#### 2.9.7. SOD-3 and MDA Examination

The core concept remains the same as in the detection of IL-6/TNF-α using Sandwich ELISA. Still, there are critical procedural differences between the Kit ELISA MDA (RAT) Abclonal RK15281 and the Kit ELISA SOD-3 (RAT) Reedbio RE2412R, which are used as reagents. The primary difference lies in the loading sequence. ABclonal often uses a “step-by-step” binding approach, while many modern kits (including some Reedbio lines) use a “simultaneous” incubation for the primary capture and detection.

Key operational differences include (1) incubation times (the “Speed” difference): ABclonal MDA is a longer assay; the total incubation time before color development is approximately 4 h. Reedbio SOD-3 follows the same timing as the TNF-α kit, with a total incubation time of approximately 3 h. (2) Reagent preparation (HRP vs. Streptavidin-HRP); ABclonal often uses a concentrated Streptavidin-HRP that requires a specific 1:100 dilution in a dedicated “HRP Diluent”. Reedbio: Usually provides a concentrated HRP conjugate. (3) Standard curves and units; MDA is usually measured in ng/mL or nmol/mL. SOD-3 is usually measured in ng/mL or pg/mL.

### 2.10. Ethical Statement

These procedures were approved by the Health Research Ethics Commission of the Faculty of Medicine, Universitas Diponegoro, Semarang, Indonesia, with protocol number 088/EC-H/KEPK/FK-UNDIP/VIII/2024. No generative artificial intelligence (GenAI) is used in this paper.

## 3. Results

In each experiment, including independent replications, we thoroughly investigated and compared the effects of 8- and 12-week exposure durations. We specifically focused on the impacts of nicotine and AA in aerosol form as opposed to traditional cigarette smoke. The analysis included a comprehensive assessment of variations in body weight, plasma levels of inflammatory markers such as IL-6 and TNF-α, and the antioxidant enzyme SOD3 and the oxidative stress indicator MDA. Furthermore, the study meticulously described and examined the pathological changes observed in the trachea-alveolar region.

**Figure 1:**
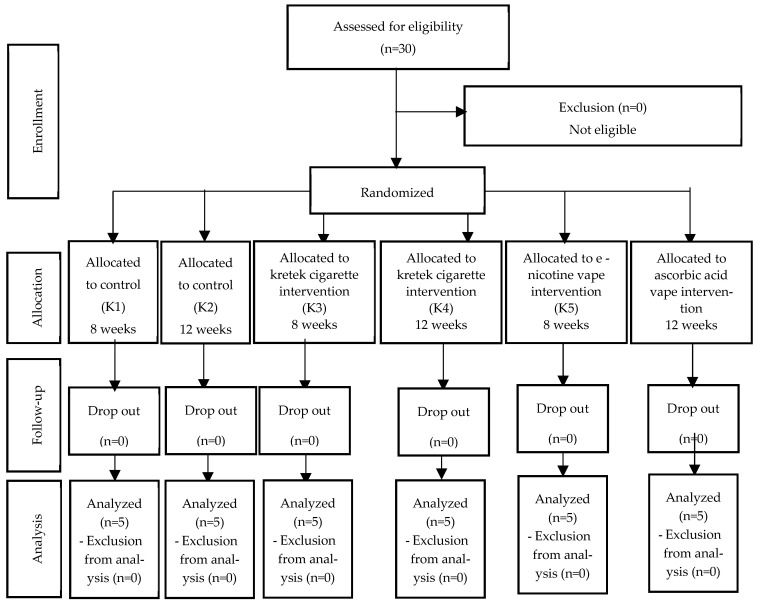
The CONSORT diagram.

In Figure 1, we present a comprehensive overview of the total enrollment process for the 30 subjects involved in the study. This figure illustrates how participants were allocated into their respective groups and the follow-up procedures implemented throughout the study, and concludes with the analysis phase. Notably, the data indicate no drop-outs or exclusions among participants, highlighting the integrity of the enrollment process and the successful retention of all subjects throughout the study.

Table 1 shows body weight changes before and after the tests for groups K1-K6. K1 had high variation after the test (SD = 44.64), with an overall SD of 44.75. K2 had stable weights before (SD = 2.30) but greater variation after (SD = 37.80), resulting in a combined SD of 37.87. K3 increased by an average of 141.0 g, but the SD rose from 4.66 to 32.10, indicating greater variability among individuals. K4 had an average increase of 32.8 g and a combined SD of 36.62, showing wide variation. K5 showed a slight average increase and a combined SD of 21.77, close to the average difference of 25.6 g. K6 had a significant average increase of 68.6 g and a combined SD of 21.79, indicating consistent changes despite more variability. These data suggest that short-term cigarette exposure (K3) did not hinder weight gain. In contrast, electronic nicotine delivery (K5) and long-term cigarette use (K4) reduced expected weight gain compared with the control groups.

There is a big difference between the highest weight gain (141.0 g) and the lowest (25.6 g). This will likely show a significant result with One-Way ANOVA (*p* < 0.05), requiring a Post Hoc test. Tukey’s test compares every pair of groups. The groups form three main “statistical clusters”. In Cluster A (normal growth: Groups K1, K2, K3), the weight difference between the control group (K1 at 138.6 g) and the group that smoked three cigarettes for 8 weeks (K3 at 141.0 g) is only 2.4 g. This difference is not significant (*p* > 0.05), indicating that smoking three cigarettes daily for 8 weeks does not significantly affect growth. Cluster B shows significant growth inhibition in the K4 and K5 groups. Group K5, using E-nicotine vapes for 8 weeks, had an average weight of 25.6 g, while Group K4, smoking cigarettes for 12 weeks, weighed 32.8 g. Both groups show a significant weight difference compared to the control group (*p* < 0.05) but are similar to each other. Cluster C shows moderate growth inhibition in Group K6, which used E-ascorbic acid vapes for 12 weeks, with an average weight of 68.6 g. This group also has a significant weight difference compared to the control (*p* < 0.05) and the E-nicotine group (K5). This suggests that the ascorbic acid vape affects growth less than the nicotine vape and long-term smoking.

Table 2 shows how cigarette smoke, e-nicotine, and e-ascorbic acid aerosols affect IL-6 and TNF-α. At 12 weeks, the cigarette group (K4) and the e-ascorbic acid group (K6) had the highest IL-6 levels: 11.45 ± 1.17 and 11.83 ± 1.56, respectively. Both groups had higher IL-6 levels than the 8-week group and the 12-week control group. There was no significant difference in IL-6 levels between K4 and K6 at 12 weeks. Overall, IL-6 levels varied significantly across all groups (F (5, 24) = 56.6, *p* < 0.001). Significant differences were observed for TNF-α (F (5, 24) = 81.1, *p* < 0.001). High-response groups may be observed in the 8-week groups K1, K3, and K5, which showed significantly higher TNF-α levels than all 12-week groups. Low-response groups were identified in the 12-week groups (K2, K4, K6), which showed a marked decrease in TNF-α concentrations compared to the 8-week cohorts.

Table 2 also presents the SOD3 levels, which varied significantly across the study (F (5, 24) = 10.08, *p* < 0.001). The highest antioxidant activity was in group K3 (12.75 ± 1.10), which was significantly higher than all 12-week groups (K2, K4, K6). The 8-week control (K1) also maintained significantly higher SOD3 levels than the 12-week cigarette (K4) and e-ascorbic acid (K6) groups. Unlike the other markers, MDA levels showed no significant variation among the groups (F (5, 24) = 0.447, *p* = 0.812), and Tukey’s test confirmed no significant pairwise differences in lipid peroxidation (MDA) across interventions or durations.

Based on the data provided in Table 3, the following is a descriptive analysis of the tracheal and alveo-pulmonary histopathology results across the six study groups. The density of goblet cells showed a statistically significant variation across groups (F (5, 24) = 3.235, *p* = 0.022). The group K3 exhibited the highest density (4.2 ± 2.44 N/mm), followed by K4 (3.93 ± 2.67 N/mm). Despite the significant ANOVA, Tukey’s HSD test revealed no specific significant pairwise differences. The difference between K3 and the K1 control was noted as borderline but did not reach the significance threshold (difference of 3.07 vs. the required 3.227).

Mucosal thickness varied significantly among the groups (F (5, 24) = 9.40, *p* < 0.001). The peak measurements were in the 8-week intervention groups, K5 (e-nicotine) and K3 (cigarette), which recorded the thickest mucosa at 71.03 ± 13.59 µ and 66.88 ± 17.92 µ, respectively. Mucosal thickness in K3 and K5 was significantly greater than in the 12-week groups, including K2 (control), K4 (cigarette), and K6 (e-ascorbic acid).

The qualitative scoring of total scores for inflammatory cell distribution and alveolar lining cell damage (Table 3) showed that the 12-week control (K2) and the 12-week e-ascorbic acid group (K6) both received the highest score of 7. In contrast, the 8-week cigarette group (K3) received the lowest score of 5. Alveolar lining cell damage was similar to the inflammation scores, with K2 and K6 showing the highest damage scores (7) and K3 the lowest (5).

**Figure 2:**
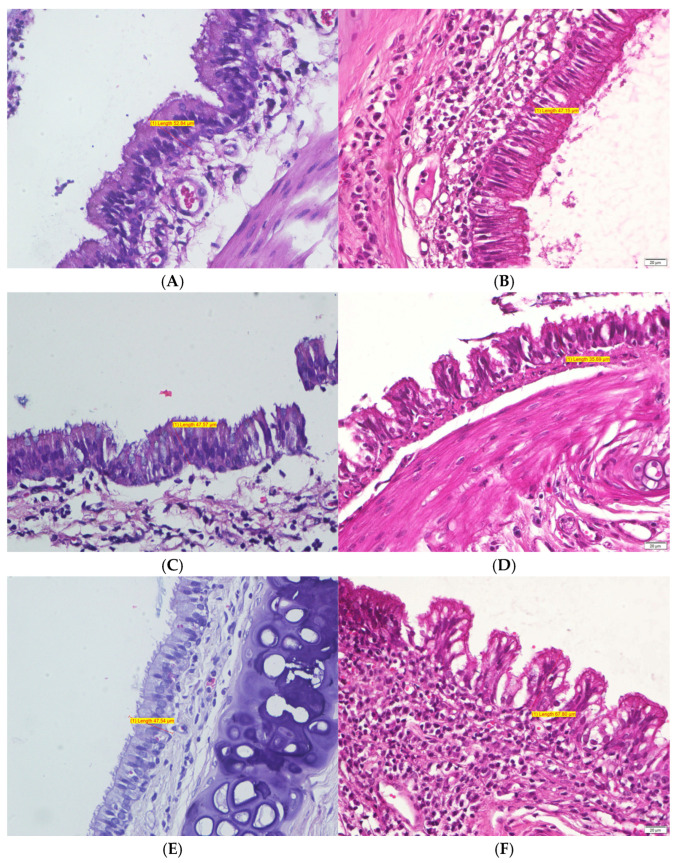
The comparative histopathology of tracheal transection between groups. Tracheal histopathology under light microscope at 400× magnification. (**A**) K1 = control 8 weeks, (**B**) K2 = control 12 weeks, (**C**) K3 = cigarette 8 weeks, (**D**) K4 = cigarette 12 weeks, (**E**) K5 = e-nicotine 8 weeks, and (**F**) K6 = e-ascorbic acid 12 weeks. Histopathological presentation of the tracheal mucosal layer across experimental groups under light microscopy (H&E staining, 400× magnification). Scale bars represent 20 µm. Structural changes indicate differences in epithelial thickness, mucosal membrane alignment, and cellular infiltration across durations (8 vs. 12 weeks) and interventions (cigarette smoke, e-nicotine, and e-ascorbic acid aerosols).

Figure 2 showed that in cigarette exposure (Figure 2C,D), goblet cell density decreased, followed by squamous metaplasia, in which the flexible ciliated epithelium is replaced by tougher, non-functional cells. The alveolar walls begin to thin and rupture (early emphysema/pre-COPD tissue remodeling). The lowest goblet cell density was observed with 8 weeks of E-nicotine exposure (C) and generally shows milder irritation. However, high concentrations of propylene glycol can cause early dehydration of the epithelial surface, resulting in e-ascorbic acid for 12 weeks (F).

**Figure 3:**
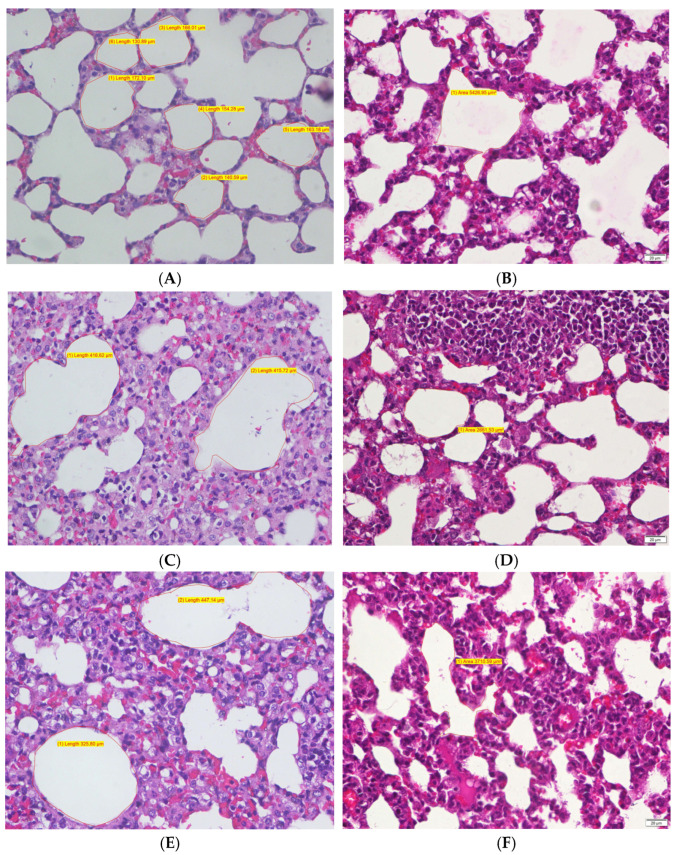
The comparative histopathology of alveo-pulmonary between groups. Alveo-pulmonary under a light microscope at 400× magnification. (**A**) K1 = control 8 weeks, (**B**) K2 = control 12 weeks, (**C**) K3 = cigarette 8 weeks, (**D**) K4 = cigarette 12 weeks, (**E**) K5 = e-nicotine 8 weeks, and (**F**) K6 = e-ascorbic acid 12 weeks. Histopathological presentation of the tracheal mucosal layer across experimental groups under light microscopy (H&E staining, 400× magnification). Scale bars represent 20 µm. Structural changes indicate differences in epithelial thickness, mucosal membrane alignment, and cellular infiltration across durations (8 vs. 12 weeks) and interventions (cigarette smoke, e-nicotine, and e-ascorbic acid aerosols).

Figure 3 shows that the alveolar walls begin to thin and rupture (early emphysema) in 3C, 3D, and 3E. The alveolar circumference of K3 (Figure 3C) > K1 (Figure 3A), K2 (Figure 3B), K4 (Figure 3E), and K6 (Figure 3F). In phytochemical intervention, e-liquid contains protective phytochemicals (ascorbic acid), and studies often show a preservation of the basement membrane and a significant reduction in the thickening of the alveolar septa (Figure 3F) compared to nicotine-only groups (Figure 3E).

## 4. Discussion

### 4.1. Body Weight Change

The link between smoking and weight loss is complex. Smokers often have a lower Body Mass Index (BMI) but a higher waist-to-hip ratio (WHR), according to Yu et al. (2018) [41]. This suggests that smoking shifts fat to the abdomen, increasing the risk of insulin resistance and metabolic syndrome, despite lower overall weight [41]. Research also suggests that while acute nicotine exposure increases metabolic rate, chronic exposure may eventually lead to a decrease in metabolic rate and an increase in appetite over very long durations, as the body adapts or as tobacco-related illnesses (like COPD) limit physical activity [42].

Smoking three cigarettes a day for 12 weeks leads to oxidative stress and reduced energy use, causing less weight gain compared to a control group and those exposed for 8 weeks. The larger weight difference in the 8-week group likely comes from body adaptation or immediate damage. While people usually gain weight as they age, those exposed to smoke gain significantly less (*p* < 0.05). Research by Chen et al. (2008) shows that weight gain is stable for the first 8 weeks but decreases by 12 weeks [43]. Smoke-exposed individuals weigh less than those in the control and “pair-fed” groups, indicating weight loss from reduced food intake and metabolic changes [43].

Cigarette smoke strongly affects the body starting around week 8, causing damage and increasing inflammation. By week 12, the body begins to adjust and lower these reactions. Smoke generates reactive oxygen species (ROS) that disrupt the body’s balance, leading to higher levels of damage markers, such as MDA, and lower antioxidant levels, such as glutathione. It also raises Uncoupling Proteins (UCPs), particularly UCP1 in brown fat and UCP3 in muscle. These proteins waste energy by dissipating it as heat rather than storing it [39].

Research shows that body weight changes depend on the substance used (e-nicotine or e-ascorbic acid) and exposure duration (8 weeks for e-nicotine or 12 weeks for traditional cigarettes). The most significant weight gain differences appear between control groups and e-nicotine users or long-term smokers. E-nicotine raises TNF-α levels, leading to tissue irritation, while long-term smoking increases IL-6 and TNF-α, which cause tissue damage and weight loss. In contrast, vaping e-ascorbic acid reduces inflammation and helps maintain weight. Overall, body weight differences reflect the struggle between smoke-induced oxidative stress and the antioxidant defense provided by ascorbic acid.

### 4.2. The Comparative Effects on IL-6 and TNF-α Levels

The data show notable differences in IL-6, TNF-α, and SOD3 levels, but not in MDA. This raises questions about which pathways are affected by specific phytochemical aerosols compared with those affected by traditional cigarette smoke. Long-term exposure (12 weeks) to cigarette smoke and e-ascorbic acid aerosol leads to higher IL-6 levels than in the 8-week groups. This suggests that long-term aerosol exposure can cause systemic inflammation [44]. After 8 weeks, the body manages the irritation well. But after 12 weeks of higher exposure, it experiences ongoing inflammation, which raises the risk of heart and metabolic diseases.

A study found that e-ascorbic acid aerosol, often seen as a healthier option, triggers an IL-6 response similar to cigarette smoke. This indicates that the way a substance is delivered, such as via aerosol, can be just as harmful as its chemical makeup. The IL-6 levels in the e-ascorbic acid group (11.83 ± 1.56) were comparable to those in the cigarette smoke group (11.45 ± 1.17). While phytochemicals usually provide protection, this study suggests that aerosolization or high ascorbic acid concentration might reduce their antioxidant benefits and possibly make them harmful in certain conditions [45,46].

At 12 weeks, the ascorbic acid group had an average IL-6 level of 11.83 ± 1.56 pg/mL, similar to that of the heavy traditional cigarette smoke group (11.45 ± 1.17 pg/mL). High IL-6 levels indicate inflammation in the body. These findings support the “General Irritant Theory,” which states that repeated inhalation of fine particles can trigger the lungs’ immune response regardless of particle composition. Components in e-liquids, such as PG and VG, can cause lung inflammation and oxidative stress even in the absence of nicotine or tobacco [47,48,49].

When both a known toxin, like cigarette smoke, and a neutral substance, such as ascorbic acid, produce the same inflammatory marker, it becomes harder to pinpoint the toxicological pathways [50]. IL-6 alone does not give the whole picture of organ damage. For example, cigarette smoke can cause DNA damage or cancer that e-ascorbic acid cannot, even if IL-6 levels are similar. However, smoking habits and inflammatory markers show that IL-6 mainly drives the long-term rise in C-reactive protein (CRP), and IL-6 levels are much higher in long-term smokers than in non-smokers [51]. Ascorbic acid, often known as vitamin C, is often used to help reduce inflammation. However, this result indicates that when delivered via heat or aerosolization, it may lose its chemical properties or fail to function effectively in the lungs. This change could lead to misunderstandings among consumers about its antioxidant benefits [52,53].

It is well-documented that TNF-α is a “first responder”. High levels at 8 weeks followed by a decline at 12 weeks align perfectly with the transition from an acute inflammatory phase to a regulatory or chronic phase [54,55]. Comparing two time points, such as 8 and 12 weeks, provides a clearer view of the immune system. It helps researchers understand how inflammation calms down. A drop in TNF-α does not always indicate improvement; the immune system might switch to other signals, such as IL-6 or IL-10. Relying only on TNF-α can be misleading [56]. Measuring TNF-α levels helps doctors determine the best time for anti-inflammatory treatments. A low level at 12 weeks may indicate immune exhaustion instead of recovery. If the underlying issue, such as an infection or injury, persists, the body might struggle to produce enough TNF-α.

The inflammatory process has three phases. In the acute phase (weeks 1–8), the body produces high levels of pro-inflammatory cytokines, such as TNF-α and IL-1β, to recruit immune cells to the site of injury. During the transition phase (weeks 8–12), the body begins to balance itself, and pro-inflammatory markers decrease. In the chronic phase (week 12 and beyond), if the issue is not resolved, the body may shift to tissue remodeling or ongoing low-grade inflammation [57,58].

### 4.3. The Comparative Effects on SOD3 and MDA Levels

The study shows a significant decrease in SOD3 levels in groups exposed for 12 weeks compared to those exposed for 8 weeks. This supports recent findings that longer exposure to aerosols increases oxidative stress, overwhelming the body’s antioxidant defenses. However, MDA levels, which indicate lipid peroxidation, did not change significantly (*p* = 0.812). This is surprising, since MDA typically increases while SOD3 decreases in chronic smoke-exposure studies. It may mean that 12 weeks was not enough to cause clear lipid damage or that the sample size was too small to detect differences.

Both cigarette and e-liquid use lead to a significant drop in SOD3 levels over time. SOD3 is an important enzyme that protects tissues from damage by free radicals. After 12 weeks, the levels for cigarette and e-liquid users were much lower (9.33 ± 0.10 and 9.07 ± 0.13) compared to the 8-week control group. This decline indicates that the body’s antioxidant system may be exhausted.

Cigarette smoke exposure over 8 weeks increases antioxidant activity via the SOD-3 enzyme, consistent with Tang et al. (2022), who reported enhanced antioxidant activity in response to greater oxidative stress during 2-week heat stress exposure [59]. Prolonged exposure to harmful substances in smoking devices, like vapes and cigarettes, decreases the antioxidant activity of the SOD-3 enzyme. Chronic smokers exhibit lower SOD levels than novice smokers due to a disrupted IL-6-mediated SOD pathway and higher cotinine levels [60]. It has been proven that the SOD level in the heavy-smoking group is lower than that of the control non-smoking group, stressing the effect of cigarette consumption on antioxidant activity and oxidative stress levels in the body [61]. Using ascorbic acid-based vape aerosol for 12 weeks reduces SOD-3 enzyme activity, likely due to chronic inflammation. The aerosol does not change SOD-3 production. Bezerra et al. (2023) noted that increased oxidative stress from inflammatory cells in asthmatic patients decreases SOD activity [62]. Other components of the aerosol, such as glycerin, propylene glycol, and flavoring, contribute to the upper airway pathway [63]. The SOD-3 levels between 8-week and 12-week exposures show no significant difference (*p* > 0.05). This suggests that different exposure periods do not affect SOD-3 expression in the control, cigarette-exposed, or vape aerosol-exposed groups, regardless of e-liquid base.

Even “phytochemical-based” aerosols like e-ascorbic acid (K6) did not prevent this decline, suggesting that the delivery method (aerosolization) or the substances themselves may still provoke a strong oxidative response that exhausts the body’s natural antioxidant pool. While the study found no statistically significant differences in MDA levels across groups (*p* = 0.812), cigarette smoke is well established in the literature as a catalyst for lipid peroxidation. General research indicates that acrolein and other aerosol particulates trigger ROS that attack cell membranes, leading to increased MDA [64]. The lack of significance in this specific study (MDA: F (5, 24) = 0.447) may be due to the short 12-week timeframe or the limited sample size, rather than a lack of a biological effect.

Using nicotine-based vape aerosol for 8 weeks raises oxidative stress, as shown by increased MDA levels, indicating that nicotine damages fats in the body. Similarly, cigarette smoke exposure for 12 weeks also raises MDA levels. In contrast, ascorbic acid-based vape aerosol used for 12 weeks leads to the lowest MDA levels, suggesting less oxidative stress. This is likely because ascorbic acid helps neutralize free radicals, offering protection and repair from oxidative damage [46,65]. The MDA levels between 8-week and 12-week exposures show no significant difference (*p* > 0.05). It can be seen that different exposure periods do not significantly affect MDA production in the control, cigarette-exposed, or vape aerosol-exposed groups across different e-liquid bases.

The “hormetic” effect found in this research suggests that the body may initially mount an adaptive defense against smoke [66]. At the 8-week peak, the highest SOD3 activity was observed in the 8-week cigarette group (K3: 12.75 ± 1.10), which was significantly higher than in all 12-week groups. This aligns with the “oxidative eustress” theory, where low-to-moderate levels of ROS initially upregulate antioxidant enzymes as a protective measure before the system becomes overwhelmed [67]. Despite exposure to nicotine and aerosols, there were no significant pairwise differences in lipid peroxidation at any time point or across interventions. This suggests that over a 12-week period, the body’s regulatory systems may still maintain membrane integrity despite a decline in SOD3 [68].

While K6 (e-ascorbic acid) had low SOD3 at 12 weeks, the use of phytochemicals in e-liquids is often explored to mitigate aerosol-induced damage. The potential benefit, based on the literature, is that exogenous antioxidants, such as ascorbic acid, can decrease lipid peroxidation following exposure. However, the study results suggest that when delivered as an e-liquid aerosol, this protective effect might be negated by an inflammatory response, as evidenced by the high IL-6 levels in K6 [69].

The study shows that both cigarette smoke and e-ascorbic acid aerosols significantly deplete antioxidant defenses (SOD3) and increase inflammation over 12 weeks. The body may attempt an early defense (seen at 8 weeks), and systemic lipid damage (MDA) may take more than 12 weeks to become significant.

### 4.4. Temporal Progression of Tracheo-Alveolar Histopathology

The respiratory system responds to inhaled insults through a sequence of structural adaptations and eventual failures. The “temporal” aspect refers to how these tissues degrade over days, weeks, or months of exposure. Acute irritation occurs on days 1–7 (Phase 1). It can be triggered by cigarette smoke, which may cause rapid-onset mucus hypersecretion and reduced ciliary beat frequency, and is accompanied by early neutrophil infiltration of the tracheal lining. Besides e-liquid aerosol exposure, propylene glycol generally causes milder irritation, though high concentrations can lead to early dehydration of epithelial surfaces [70].

In the 4- to 12-week exposure period, the phase 2 cellular response, including chronic remodeling, occurs. Tracheal changes occur with cigarette smoke exposure, leading to squamous metaplasia, in which the flexible, ciliated epithelium is replaced by tougher, non-functional cells. Besides the alveolar destruction shown in the deep lung, where the alveolar walls begin to thin and rupture (early emphysema/pre-COPD tissue remodeling). If the e-liquid contains protective phytochemicals, studies often show preservation of the basement membrane and a significant reduction in alveolar septal thickening compared with nicotine-only groups [71].

This study revealed significant microstructural changes in tracheal–alveolar tissue. With a shorter exposure (8 weeks) to cigarette smoke, goblet cell density is higher than at 12 weeks. This means that the peak of acute exposure, the point of no return, occurred during the 8-week period, followed by irreversible injury that may decrease goblet cell density. Histopathological examination of the tracheal mucosal thickness also revealed this phenomenon. Unfortunately, differences in the distribution of inflammatory cells or in damage to alveolar lining cells were not measured.

Cigarette smoke exposure in an 8-week period has the highest goblet cell counts, altering the upper airway structure composition, marked by the higher number of goblet cells in smokers compared to ex-smokers and never-smokers. Goblet cell counts change, accompanied by changes in mucin expression, suggesting that mucin production is likely influenced by goblet cell densities [72]. Besides goblet cell hyperplasia, cigarette smoke exposure also caused goblet cell metaplasia, both of which eventually lead to hypersecretion of mucus, which causes respiratory problems [73,74]. The 8-week control group shows the lowest goblet cell counts, indicating that exposure to irritants would eventually affect tracheal structural composition, including goblet cell counts. The non-smokers group has lower goblet cell counts and higher ciliated cell counts than the smoker group, accompanied by better pulmonary functions [75]. The GCD between 8-week and 12-week exposures shows no significant difference (*p* > 0.05). It can be seen that different exposure periods do not significantly affect Goblet Cell Counts in the control, cigarette-exposed, or vape aerosol-exposed groups across e-liquid bases.

Exposure to nicotine-based vape aerosol for 8 weeks leads to a thickened mucosal epithelial layer, supporting previous findings that vape aerosol acts as an irritant, causing overproduction of respiratory mucus [76]. Normally, when the trachea is inflamed and under oxidative stress, it thickens the mucosal epithelium; however, in some cases, chronic exposure to irritants can reduce mucosal epithelial thickness. Cigarette smoke exposure over 12 weeks results in the thinnest mucosal epithelial thickness, consistent with a previous study indicating that cigarette smoke and vape aerosol accumulation could promote cell death, disrupt the protective barrier, and alter epithelial structure [77]. Exposure to cigarette smoke over a longer period alters the mucosal epithelium to a cuboidal structure with shortened, decreased cilia, reducing mucosal epithelial thickness in the trachea [78]. The thickness of the mucosal epithelium significantly differs between 8-week and 12-week exposure to irritants (*p* < 0.05). The length of exposure affects the thickness of the mucosal epithelial layer. The Bonferroni Post Hoc test shows a notable impact of exposure time in both the cigarette smoke group and the vape aerosol group. Longer exposure to these irritants increases the thickness of the tracheal mucosal epithelium, often leading to more mucus production in the upper respiratory tract [79]. The structure of the tracheal mucosal epithelium is significantly altered by smoke exposure, which is positively correlated with tracheal abnormalities, including damaged goblet cells, altered mucosal architecture, and ciliary loss [80]. Alterations in the tracheal mucosal epithelium could reduce the effectiveness of respiratory tract defenses, making it easier for pathogens to enter the body through the airways.

### 4.5. The IL-6/TNF-α Molecular Signaling Axis

The IL-6 and TNF-α axis serves as the “alarm system” of the mucosal airways, triggering chronic inflammation when elevated. Cigarette smoke activates the NF-κB pathway, which promotes 1) TNF-α release from alveolar macrophages, recruiting immune cells and inducing lung tissue apoptosis, and 2) IL-6, which facilitates the shift from acute to chronic inflammation. High IL-6 levels are linked to greater fibrosis. The data reveal a distinct pattern in IL-6 and TNF-α levels with long-term exposure. IL-6 is significantly higher in the K4 (cigarette, 12 weeks) and K6 (e-ascorbic acid, 12 weeks) groups than in controls and the 8-week groups, indicating a stronger chronic inflammatory response at 12 weeks. Conversely, TNF-α levels are higher in the 8-week groups (K1, K3, K5) than in the 12-week groups (K2, K4, K6), suggesting a shift from an acute inflammatory phase (TNF-α-dominant) to a chronic phase (IL-6-dominant) with increasing exposure duration.

The activation of the inflammatory axis often leads to systemic metabolic changes, as reflected in the weight-gain data. Groups with elevated inflammatory markers (specifically K4, K5, and K6) show significantly lower weight gain (Δ Mean) than the controls (K1, K2). While the controls (K1, K2) gained approximately 137–138 g, the 12-week cigarette group (K4) gained only 32.8 g. This suppression of weight gain correlates with the peak IL-6 levels shown in Table 2, suggesting that chronic inflammation may inhibit normal growth or cause metabolic cachexia.

The signaling axis also correlates with the depletion of the body’s antioxidant capacity. As IL-6 levels rise in the 12-week groups (K4 and K6), Superoxide Dismutase 3 (SOD3) levels drop significantly compared with the 8-week cigarette group (K3). The reduction in SOD3 at K4 and K6, alongside elevated IL-6, suggests that the inflammatory axis contributes to an environment in which oxidative stress is not effectively neutralized, potentially leading to further cellular damage.

### 4.6. Comparative Impact of Phytochemical-Based Aerosols

The data highlight the difference between chronic exposure to cigarette smoke and plant-based aerosols. Plant compounds can reduce inflammation and protect cells, while cigarette smoke raises IL-6 and TNF-α levels, leading to ongoing inflammation and damage to the respiratory lining. This results in “sloughing,” where the protective cell layer breaks down. Phytochemical e-liquids act as a buffer; they stabilize or reduce harmful markers over time, suggesting they lessen the negative effects of smoke exposure. They activate the NRF2 pathway, helping cells produce more antioxidants and become stronger. This strengthening helps maintain healthy epithelial tissue. While cigarette smoke causes damage, the phytochemical group helps maintain “tight junctions,” which hold cells together and protect the barrier. Thus, phytochemical aerosols not only reduce inflammation but also fortify the respiratory system against environmental stress.

Nicotine and ascorbic acid in aerosols can affect the body’s signaling pathways. These aerosols may reduce ROS and help prevent severe inflammation compared to traditional smoking. Timing is important for treatment; targeting the IL-6/TNF-α axis early with these phytochemicals could delay the onset of serious lung damage, such as emphysema. However, “less harmful” does not mean “safe,” and we are still studying the long-term effects of inhaling these aerosols in people. However, heating ascorbic acid causes thermal degradation into products like dehydroascorbic acid and oxalic acid, which likely act as the underlying drivers behind the pro-oxidant/pro-inflammatory spikes observed in our results.

### 4.7. Unanswered Questions and Future Perspectives

This study shows a link between aerosol exposures, lung tissue changes, and protein release in the body. However, we still have important questions. First, while our 8-week and 12-week results show a shift from short-term inflammation to long-term lung damage, we do not know if these effects improve after exposure ends. Future research should investigate whether issues such as tracheal thinning and early emphysema resolve on their own. Second, while we measured proteins such as IL-6 and TNF-α, we need to examine specific lung pathways in more detail using techniques such as Western blotting or immunostaining.

Looking ahead, the trend of “wellness vaping” requires thorough safety studies. Future research should compare different herbal- and vitamin-based vaping products across various heating settings to identify potential harmful byproducts. It also needs studies measuring how well people can clear mucus from their noses and the amount of nitric oxide in their breath when using electronic nicotine delivery systems (ENDS) and other aerosolizers. These steps are essential for providing solid evidence to help regulate alternative smoking devices.

### 4.8. Limitations

The study has several limitations related to its methods, duration, and biological markers. First, the sample size might be too small to detect important differences in lipid damage. Second, the 12-week duration may not be long enough to observe lasting lipid damage or to understand the development of long-term tobacco-related diseases, such as early emphysema. Lastly, the study lacks specific cellular data, so its findings on tissue damage cannot be confirmed by assessments of inflammatory cells or damage to the air sac lining.

Clove cigarettes, or kretek, contain unique chemicals like eugenol and have higher tar-to-nicotine ratios than regular cigarettes. While this is important for local public health, we should be careful about applying these findings globally, where clove-free cigarettes are common. Our study examined two timeframes (8 and 12 weeks) to assess short-term and long-term effects. However, this approach does not provide a detailed view of these changes over time. Future research should use shorter intervals (like 4, 6, and 10 weeks) to better understand how e-liquid causes lung inflammation.

This study takes a different approach to track changes over time, focusing on key moments when tissue changes are significant. We did not analyze the chemical structure of vape aerosol. Our findings show that inhaling e-ascorbic acid triggers an inflammatory response similar to that induced by cigarette smoke, suggesting that heating alters its antioxidant effects. We also did not measure substances formed during heating, such as dehydroascorbic acid. Future research should link these substances to lung damage. We lacked a control group using only the vehicle (Propylene Glycol/Vegetable Glycerin). Since nicotine and ascorbic acid were mixed in a 50:50 PG/VG blend, we cannot determine whether changes in the trachea and cytokine levels came from nicotine, ascorbic acid, or the carrier’s breakdown products. This limits our ability to assess the toxicity of each component and underscores the need for more studies with clear controls.

Using only IL-6 or TNF-α to measure inflammation gives an incomplete view of organ damage. Although IL-6 levels may be similar between substances like e-ascorbic acid and cigarettes, this does not consider other harm, such as DNA damage or cancer. A drop in TNF-α after 12 weeks might indicate immune exhaustion instead of healthy inflammation. There was also no notable change in MDA levels among the groups. In chronic smoke-exposure studies, it is unusual to see MDA levels drop while antioxidant defenses decrease. The drop in TNF-α at 12 weeks indicates a shift from acute to chronic conditions, but the immune system may be adjusting other cytokines that were not measured, such as IL-1β, IL-8, or IL-10. Future studies should track a wider range of cytokines over time to understand how the body recovers. A 12-week exposure in rodents can cause permanent lung damage, such as early emphysema, but it only reflects the early stages of lung disease. This timeline does not fully reflect the severity of human symptoms. Longer studies are needed to better understand advanced lung problems.

The research highlights risks to delivery methods, as some substances, such as ascorbic acid, can change from anti-inflammatory to pro-oxidant when heated or aerosolized. Additionally, the long-term effects of inhaling aerosolized phytochemicals in humans remain largely unknown.

## 5. Conclusions

This research shows that traditional cigarette smoke and e-liquid aerosols, including those with natural ingredients such as ascorbic acid, cause significant inflammation and reduce antioxidant levels over time. The body’s response shifts from a short-term defense at 8 weeks, marked by an increase in TNF-α, to a long-term inflammatory state at 12 weeks, indicated by a rise in IL-6. Initially, the body increases antioxidant activity to protect against oxidative stress, but prolonged exposure depletes these defenses, including SOD3. The study also supports the idea that a substance’s form (aerosol) can be as harmful as its chemical composition; even seemingly “neutral” substances like propylene glycol and vegetable glycerin can trigger immune responses.

Long-term exposure to harmful substances can damage the lungs, reduce goblet cell numbers, and thin the tracheal lining. This harms the body’s respiratory defenses and slows metabolism, causing inflammation marked by elevated IL-6 levels and reduced weight gain, suggesting that these substances can hinder growth. Interestingly, while ascorbic acid is an antioxidant, inhaling it as an aerosol raises IL-6 levels similarly to cigarette smoke, suggesting it may become harmful in aerosol form. Despite these risks, some plant-based aerosols may offer better lung protection.

Finally, the study establishes that “less harmful” does not mean “safe”. Body weight differentiation and tissue damage serve as visible markers of the internal struggle between oxidative stress and the body’s inflammatory defense system. The novelty of this research lies in its temporal analysis of the body’s transition from acute to chronic injury and its investigation of the paradoxical effects of aerosolized antioxidants.

## Figures and Tables

**Table 1:** The mean ± SD of body weight differences pre- and post-tests between groups.

Groups	Pre-Test	Post-Test	(Δ) Mean ± SD (g)	Sig. *p*	Tukey’s Post Hoc
	Mean ± SD (g)	Mean ± SD (g)			
K1 (*n* = 5)	168.8 ± 3.11	307.4 ± 44.64	138.6 ± 44.75	<0.001	a
K2 (*n* = 5)	165.4 ± 2.30	302.8 ± 37.80	137.4 ± 37.87	<0.001	a
K3 (*n* = 5)	165.8 ± 4.66	306.8 ± 32.10	141.0 ± 32.44	<0.001	a
K4 (*n* = 5)	179.8 ± 13.55	212.6 ± 34.02	32.8 ± 36.62	<0.001	b
K5 (*n* = 5)	169.6 ± 10.06	195.2 ± 19.31	25.6 ± 21.77	<0.001	b
K6 (*n* = 5)	171.8 ± 6.69	240.4 ± 20.74	68.6 ± 21.79	<0.001	c

Notes: K1 = control 8 week, K2 = control 12 week, K3 = cigarette 8 week, K4 = cigarette 12 week, K5 = e-nicotine 8 weeks, K6 = e-ascorbic acid 12 weeks. One-way ANOVA: F (5, 24) = 10.22, *p* < 0.001. Tukey’s post hoc test (α = 0.05): a = significantly different from K4 and K5; b = significantly different from K1, K2, and K3; and c = significantly different from K4 and K5, but not significantly different from K1, K2, or K3.

**Table 2:** The comparison of IL-6, TNF-α, SOD3, and MDA plasma levels between groups.

Groups	Variables (Mean ± SD)			
	IL-6 ^(a)^	TNF-α ^(b)^	SOD3 ^(c)^	MDA ^(d)^
K1 (*n* = 5)	5.73 ± 0.87	77.2 ± 9.98	11.85 ± 1.03	154.30 ± 64.24
K2 (*n* = 5)	8.43 ± 0.88	18.76 ± 2.48	10.06 ± 0.77	219.20 ± 83.04
K3 (*n* = 5)	6.4 ± 0.69	76.9 ± 9.44	12.75 ± 1.10	198.47 ± 119.30
K4 (*n* = 5)	11.45 ± 1.17	21.97 ± 4.33	9.33 ± 0.10	243.15 ± 106.00
K5 (*n* = 5)	6.5 ± 1.14	66.1 ± 9.80	11.05 ± 2.81	243.89 ± 242.66
K6 (*n* = 5)	11.83 ± 1.56	18.19 ± 1.62	9.07 ± 0.13	178.23 ± 53.30

Notes: K1 = control 8 week, K2 = control 12 week, K3 = cigarette 8 week, K4 = cigarette 12 week, K5 = e-nicotine 8 weeks, K6 = e-ascorbic acid 12 weeks. One-way ANOVA: IL-6: F (5, 24) = 56.6, *p* < 0.001; TNF-α: F (5, 24) = 81.1, *p* < 0.001; SOD3: F (5, 24) = 10.08, *p* < 0.001; MDA: F (5, 24) = 0.447, *p* = 0.812 (not significant). Tukey’s Post Hoc Test (α = 0.05): ^(a)^ IL-6: K4 and K6 are significantly higher than K1, K2, K3, and K5 (*p* < 0.05). No significant difference between K4 and K6 (*p* > 0.05). ^(b)^ TNF-α: K1, K3, and K5 are significantly higher than K2, K4, and K6 (*p* < 0.05). ^(c)^ SOD3: K3 is significantly higher than K2, K4, and K6 (*p* < 0.05). K1 is significantly higher than K4 and K6 (*p* < 0.05). ^(d)^ MDA: No significant pairwise differences (*p* > 0.05 for all comparisons).

**Table 3:** Comparison of tracheo-alveo-pulmonary histopathological findings between groups.

Groups	Tracheal Histopathology		Alveo-Pulmonary Histopathology		
	Goblet Cells Density ^(a)^ Mean ± SD (N/mm)	Mucosal Thickness ^(b)^ Mean ± SD (µ)	Alveolar Circumference ^(c)^ Mean ± SD (µ)	Total Score of the Distribution of Inflammatory Cells	Total Score Damage of the Alveolus Lining Cells
K1 (*n* = 5)	1.13 ± 0.52	52.40 ± 2.63	202.03 ± 29.38	6	6
K2 (*n* = 5)	1.37 ± 0.72	45.50 ± 7.48	273.58 ± 45.28	7	7
K3 (*n* = 5)	4.2 ± 2.44	66.88 ± 17.92	469.77 ± 91.31	5	5
K4 (*n* = 5)	3.93 ± 2.67	34.65 ± 6.55	221.17 ± 47.64	6	6
K5 (*n* = 5)	1.73 ± 1.05	71.03 ± 13.59	362.99 ± 51.2	6	6
K6 (*n* = 5)	2.89 ± 1.18	42.10 ± 6.54	261.61 ± 58.56	7	7
Variable					
F (df)	F (5, 24) = 3.235	F (5, 24) = 9.40	F (5, 24) = 16.72		
*p*-value	*p* = 0.022 *	*p* < 0.001	*p* < 0.001		
Tukey’s HSD (α = 0.05)	No significant pairwise differences (K3 vs. K1 borderline)	K3, K5 > K4, K2, K6	K3 > K1, K2, K4, K6; K5 > K1, K4		

Notes: K1 = control 8 week, K2 = control 12 week, K3 = cigarette 8 week, K4 = cigarette 12 week, K5 = e-nicotine 8 weeks, K6 = e-ascorbic acid 12 weeks. One-way ANOVA: ^(a)^ Goblet cells density (N/mm): F = 3.235, * *p* = 0.022 (*p* < 0.05, significant). ^(b)^ Mucosal thickness (µ): F = 9.40, *p* < 0.001 (F critical 2.62). ^(c)^ Alveolar circumference: F (5, 24) = 16.72, *p* < 0.001. Tukey’s post hoc test: ^(a)^ Goblet cells density = Significant if the difference in mean > 3.227: • K3 vs. K1 (4.20 − 1.13 = 3.07) → ✗ (no significant difference, although it is close). • K3 vs. K2 (2.83) ✗; K3 vs. K5 (2.47) ✗; K4 vs. K1 (2.80) ✗; K4 vs. K5 (2.20) ✗; K6 vs. K1 (1.76) ✗. ^(b)^ Mucosal thickness = Significant if the difference in mean > 20.41: • K3, K5 > K4, K2, K6. • K5 vs. K1 (18.63) ✗; K3 vs. K1 (14.48) ✗. ^(c)^ Alveolar circumference = Significant if the difference in mean is >20.41. K3 is significantly higher than K1, K2, K4, and K6 (*p* < 0.05), and K5 is significantly higher than K1 and K4 (*p* < 0.05). K3 vs. K5: *p* = 0.051 (not significant, but borderline). No other significant differences (*p* > 0.05).

## Data Availability

The data presented in this study are available at https://drive.google.com/drive/folders/1hDxqLrGeTYMl2iSPboK4himGbllNSoUi?usp=sharing (accessed on 12 March 2026).

## Abbreviations

The following abbreviations are used in this manuscript:

IL-6	Interleukin-6
TNF-α	Tumor Necrosis Factor-Alpha
IL-1β	Interleukin-1-beta
IL-8	Interleukin-8
SOD-3	Superoxide Dismutase-3
MDA	Malondialdehyde
AA	Ascorbic acid
NMC	Nasal Mucociliary
ARRIVE	Animal Research: Reporting In Vivo Experiments
PG	Propylene Glycol
VG	Vegetable Glycerin
AVMA	American Veterinary Medical Association
GCD	Goblet Cells Density
ANOVA	Analysis of variance
H&E	Hematoxylin and Eosin
EDTA	Ethylenediaminetetraacetic Acid
IACUC	Institutional Animal Care and Use Committee
COPD	Chronic Obstructive Pulmonary Diseases
OD	Optical Density
BMI	Body Mass Index
WHR	Waist-to-hip ratio
UCP	Uncoupling Protein
CRP	C-reactive Protein
